# Assessment of quality of antenatal care services in Nigeria: evidence from a population-based survey

**DOI:** 10.1186/s12978-015-0081-0

**Published:** 2015-09-18

**Authors:** Adeniyi Francis Fagbamigbe, Erhabor Sunday Idemudia

**Affiliations:** School of Research and Postgraduate School (SoRPS), North West University, Mafikeng, South Africa; Department of Epidemiology and Medical Statistics, Faculty of Public Health, College of Medicine, University of Ibadan, Ibadan, Nigeria

**Keywords:** Quality antenatal care, Tetanus toxoid injection, Iron supplementation, Intermittent preventive treatment, Intestinal preventive drug

## Abstract

**Background:**

The aim of the newly introduced “focused Antenatal Care (ANC)” is not only to achieve a minimum number of 4 visits, but also the timeliness of the commencement of the visits as well as the quality and relevance of services offered during the visits. This study is therefore designed to assess the quality of ANC services in Nigeria.

**Methods:**

We used information supplied by the 13410 respondents who claimed to have used the ANC facilities at least once within five year preceding the 2013 Nigeria Demographic and Household Survey (NDHS). Ten components of ANC including: offer of HIV test, Tetanus Toxoid injection, receiving iron supplementation, intermittent preventive treatment (IPT), intestinal preventive drug (IPD), timely ANC enrollment and number of visits were assessed. Receipts of all the ten components were classified as desirable (good) quality of ANC services while receipt of eight critical components among the ten were assumed to be the minimum acceptable quality. Data was weighted and analyzed using descriptive statistics and logistic regression models at 5 % significance level.

**Results:**

Measurement of blood pressure and receiving iron supplementation were the most commonly offered ANC component in Nigeria with 91.0 % each while IPD and IPT were given to only 20.7 % and 37.6 % respectively. Less than two thirds were taught on PMTCT while 41.7 % had HIV test and obtained results. Only 4.6 % (95 % CI: 4.2–5.1) of women received good quality of ANC while nearly 1.0 % did not receive any of the components. About 11.3 % (95 % CI: 10.6–11.9 %) of the attendees had minimum acceptable quality of ANC. Receipt of good quality ANC services was higher among users who initiated ANC early, had at least 4 ANC visits, attended to by skilled health workers, attended government and private hospitals and clinics. Higher odds of receiving good quality of ANC were found among users who lives in urban areas, having higher educational attainment, belonging to households in upper wealth quintiles and attended to by skilled ANC provider.

**Conclusions:**

The levels of desirable and minimum acceptable quality of ANC services were poor in Nigeria thereby jeopardizing efforts to achieve the MDGs. There is need for intensified commitment by national and state governments in Nigeria as well as other stakeholders to ensure that main components of ANC are received by the users.

## Introduction

The essence of antenatal care (ANC) is to prepare women for birth and parenthood and prevent problems for pregnant women, mothers and babies through early detection, alleviation and or management of health problems that affect mothers and babies during pregnancy [1]. The success of any ANC depends on its policy formulation and implementation [2]. It also depend on functional and operational continuum of care with affordable, accessible, high quality care during and after pregnancy and childbirth [1, 3, 4]. For ANC programme to be effective, important components of ANC must be provided [1, 2, 4–6]. Inadequate ANC, both in terms of coverage and quality,  has been associated with adverse pregnancy outcomes [3]. Although maternal mortality ratio (MMR) is impacted by many causes including obstetric, social, cultural and economic factors, adequate use of ANC could contribute to reduction of the ever high MMR in Nigeria [4, 7]. Nigeria with MMR of 560 per 100000 compared to global average of 210 [8] undoubtedly needs an improved ANC coverage as well as high quality ANC service delivery. Trends in ANC use worldwide, especially as it affects developing countries, prompted the WHO to define a new ANC model, “focused ANC”, based on four goal-oriented visits [9]. This model was further broken into what services are rendered in each visit [1–3] and emphasized minimum of four visits and what must be done in each of the visits. Literature is replete on the fact that ANC coverage has improved in Nigeria [8, 10–13]. This has been attributed to free ANC services  rendered in some parts of the country [11, 13]. The pertinent question is if “Does  the increased ANC coverage translates to effective and quality ANC service delivery in Nigeria?”

While increased ANC coverage is a welcome development, ANC coverage alone cannot guarantee success of ANC services. Besides increase in coverage of ANC services, provision of quality ANC services will have the greatest impact on women accessing these services. It is not sufficient for a pregnant woman to visit ANC facility; she must meet minimum requirements and be offered necessary components of ANC. Although there is no consensus on the indicators for quality of ANC care [1], it may include early initiation and having four or more ANC visits and coverage of essential interventions delivered through ANC services [1, 4, 14]. Skill of ANC providers, staff motivation, budgetary provisions, integration with other health programmes and availability of consumables, drugs and basic equipment can seriously impact on the quality of ANC services [3, 15–19]. A recently concluded study in Nepal found that good quality ANC was higher for women attended to by skilled providers. The study also noted that those that made at least four visits had health education, took iron supplements, had Tetanus Toxoid injections and had blood pressure taken [20]. Similarly, a Nigerian study [21] found that most ANC users had four or more visits; high provision of malaria prophylaxis, iron and folate supplements but low hemoglobin estimation and Tetanus Toxoid injections. In this study, we hypothesized that improved ANC coverage in Nigeria could have impacted a good quality of ANC services and that quality of ANC services received could vary across the characteristics of the women.

Several studies [2, 4, 5, 19, 22–24] have determined factors affecting ANC use but only few [25–27] have exploited the quality of services rendered at various antenatal clinics in Africa. In Nigeria, the few studies [21, 27–29] on quality of ANC in Nigeria so far have been geographically restricted. For instance, some studies on ANC quality [21, 27] were carried out in South West Nigeria, in South East [28] and a 2007 study in North Central Nigeria [29]. The present study, apart from using a nationally representative data which covered the entire country also has a large sample size. The objectives of this study are to assess the quality of ANC services in Nigeria. The study was also aimed at determining if the improved ANC coverage in Nigeria could have impacted good quality ANC deliveries. We also identified women characteristics influencing receipt of good quality of ANC with a view of informing stakeholders in ANC programming and funding.

## Methods

We used the data from the 2013 Nigeria Demographic and Health Survey (NDHS) [11]. The sample was nationally representative and population-based. The respondents were selected using a stratified three-stage cluster design. First was selection of 904 clusters, 372 in urban areas and 532 in rural areas. At the second stage, 45 households were selected per cluster from which a minimum target of 943 completed interviews per state involving women aged 15–49 were sampled at the third stage. A representative sample of 40,680 households was selected for the survey. At the end, 39,902 women age 15–49 years were identified of which 98 % were validly interviewed. Other details of the survey methodology have been reported in 2013 NDHS [11].

Our analysis was based on the records of 13410 (66.1 %) women who have visited an ANC facility at least once among all women who gave birth in the five years preceding the survey. The most recent births were referred for all women.

In this study, we assessed the 10 nationally recommended and recognized components of ANC in Nigeria, they are receiving of iron supplements, intestinal parasite drugs (IPD), at least two doses of Tetanus Toxoid injections, malaria intermittent preventive treatment in pregnancy (IPTp) and health education on danger signs and complications during pregnancy; blood pressure measurement; urine tests; blood tests; health talk on prevention of mother to child transmission (PMTCT) of HIV/AIDS and HIV/AIDS counselling, testing and collection of results. These components have also been either recommended or recognized earlier [1, 9, 20]. The outcome variable in this study was the quality of ANC services. We assessed the quality of ANC in terms of the desirability and minimum acceptable levels. We defined desirable (good) quality of ANC as the receipt of all the 10 components as used in previous study [3] while the receipt of the eight most critical of the components were defined to be minimum acceptable level. The two components that we considered less critical are receiving of intestinal parasite drugs (IPD) and health talk on prevention of mother to child transmission (PMTCT) of HIV/AIDS.

The independent variables were socio-demographic characteristics of the respondents (residence: rurality and geographical, women’s age at child birth, parity, women’s household wealth quintile, women’s educational attainment), skill of the person and institution providing ANC, the number of ANC visits, the timing of the first ANC visit and decision-making power. Using the definition of “skilled health worker” by WHO, “Doctors”, “Nurse/Midwife” and “Auxiliary Nurse/Midwife” were grouped as “skilled ANC provider” [1, 9, 11] while others were grouped as “unskilled” ANC provider. The skilled providers are the only health professionals authorized to provide ANC in Nigeria.

### Ethical considerations

Ethical approvals for the study was sought and obtained from the National Health Research Ethics Committee and assigned number NHREC/01/01/2007 as earlier documented [11].

### Statistical analyses

We used logistic regression to model relationship between the good quality of ANC and independent variables at the bivariate level. Significant variables at bivariate levels were included in a multiple logistic regression model and were adjusted for. Logistic regression model of the form

$$ f\left({y}_i\right)= ln\frac{P\left({y}_i\right)}{1-P\left({y}_i\right)}={\beta}_0+{\beta}_1{x}_{i1}+\dots \dots .+{\beta}_k{x}_{ik} $$

determines the association between a dichotomic dependent variable and independent variables by converting the dependent variable to probability scores taking on values between zero and one

$$ P\left({y}_i\right)=\frac{e^{\beta_0+{\beta}_1{x}_{i1}+\dots \dots .+{\beta}_k{x}_{ik}}}{1+{e}^{\beta_0+{\beta}_1{x}_{i1}+\dots \dots .+{\beta}_k{x}_{ik}}}=\frac{1}{1+{e}^{-\Big({\beta}_0+{\beta}_1{x}_{i1}+\dots \dots .+{\beta}_k{x}_{ik\Big)}}} $$

where βj, y_i_ and x_*ij*_ are the coefficients, dependent variable and independent variables respectively Hosmer and Lemeshow statistic and Omnibus tests were used to check the fitness of the models. All analyses were performed at 5 % significance level using sampling weights.

## Results

The mean age of the 13,410 women who had used ANC services within the five years preceding the survey was 29.7 ± 7.1 years. Nearly half, 6131(45.7 %) of the respondents were aged 20–29 years. Majority, 10925(81.5 %) had at least four ANC visits, 12165(90.7 %) received care from skilled ANC providers while 9339(69.6 %) received care from government hospitals and clinics as shown in Table 1.

**Table 1:** Distribution of socio-demographic characteristics and health seeking behavior of the Respondents

Characteristics	*n* = 13410	Percent
Residence		
Urban	6462	48.2
Rural	6948	51.8
Educational attainment		
No formal education	4080	30.4
Primary	3083	23.0
Secondary	4991	37.2
Higher	1256	9.4
Marital status		
Never married	426	3.2
Married or LWSP	12511	93.3
Formerly married	473	3.5
Age (Mean,SD) (29.7,7.1 yrs)		
<20	705	5.3
20–29	6131	45.7
30–39	5119	38.2
40+	1455	10.9
Wealth quintile		
Poorest	1406	10.5
Poorer	2365	28.1
Average	2895	21.6
Wealthier	3277	24.4
Wealthiest	3467	25.9
Geographical zone		
North Central	2125	15.9
North East	2018	15.1
North West	3275	24.4
South East	1630	12.2
South South	1566	11.7
South West	2796	20.9
Birth order		
1	2614	19.5
2	2349	17.5
3	2077	15.5
4	6370	47.5
Timing of ANC Initiation		
1^st^ Trimester	3598	26.9
2^nd^ Trimester	8310	62.2
3^rd^ Trimester	1453	10.9
Number of ANC Visits		
Less than 4 times	2485	18.5
4 times or more	10925	81.5
Participation in decision making		
Yes	3803	28.4
No	9607	71.6
Skill of ANC provider		
Skilled	12165	90.7
Unskilled	1245	9.3
Place of receiving ANC		
Homes	377	2.8
Govt hospital/clinics	9339	69.6
Govt others	465	3.5
Private Hospital/Clinic	3032	22.6
Others	197	1.5

**Fig. 1:**
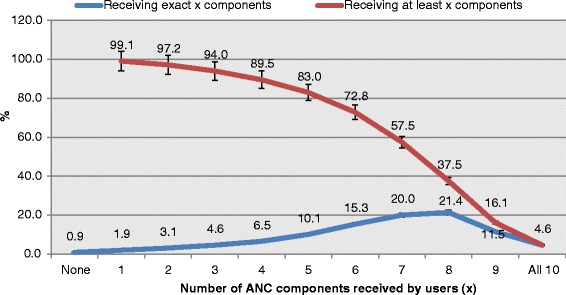
Distribution of ANC components received by the users

Blood pressure measurement is the most offered component 12186(90.9 %) (95 % CI: 90.4–91.4 %) and followed closely by receiving Iron supplements 12172(90.8 %). The least offered components of ANC were use of IPD and IPTp with 2769(20.7 %) and 5047(37.6 %) respectively. Less than two thirds, 8446(63.0 %), were educated on PMTCT of HIV/AIDS while 5591(41.7 %) had HIV test and knew their status. Only 4.6 % (95 % CI: 4.2–5.1 %) of the ANC service users had received all the 10 components and therefore had desirable quality of ANC (Table 2). About 11.3 % (95 % CI: 10.6–11.9 %) received the eight critical components and thus had minimum acceptable quality of ANC. While 11.7 % (95 % CI: 15.0–18.1 %) received nine of the ten components, only 5029(37.5 %) received at least eight of the ten components. Almost a percent of all the ANC services users did not receive any of the 10 components of ANC (Fig. 1). Further analysis showed that nearly half (49.5 %) of those that received none of the ten components attended “homes” while 31.1 % visited “government hospitals and clinics” (not shown in the tables).

**Table 2:** Components of antenatal care received during last pregnancy

ANC Components (*N* = 13410)	Number	Percent	95 % CI
Blood pressure taken*			
Yes	12186	90.9	90.4–91.4
Had iron tab/syrup*			
Yes	12172	90.8	90.3–91.3
Blood sample taken*			
Yes	11003	82.1	81.3–82.8
Urine sample taken*			
Yes	10979	81.9	81.1–82.6
Had 2 or more tetanus injection*			
Yes	9736	72.6	71.7–73.5
Told danger signs*			
Yes	9031	67.4	66.4–68.3
Told about PMTCT			
Yes	8446	63.0	62.0–64.0
Had HIV test & obtained HIV test result*			
Yes	5591	41.7	40.4–43.0
Had malaria drug for IPT*			
Yes	5377	40.1	39.2–41.2
Had intestinal parasite drugs			
Yes	2769	20.7	19.1–22.2
Had all the 8 critical components			
Yes	1487	11.3	10.6–11.9
Had all 10 components			
Yes	567	4.6	4.2–5.1

*Critical components

A higher proportion of ANC users from urban areas received desirable quality of ANC, compared to those from rural area 422(6.5 %) vs 197(2.8 %). Respondents with higher education had highest level of desirable quality of ANC 80(6.3 %) compared to 139(3.4 %) users without formal education. Proportion of ANC users receiving desirable quality ANC services reduced with wealth status from 222(6.4 %) among users in wealthiest quintile to 26(1.8 %) in the poorest quintile. Receipt of good quality ANC services was lowest among users who commenced ANC attendance during the third semester. Higher proportion of users who had at least 4 ANC visits received good quality ANC services than those who attended less than 4 times. On regional bases, attendees from North West zone had highest (9.5 %) level of desirable quality of ANC followed by South East (4.4 %). Having minimum acceptable quality of ANC was more prevalent among pregnant women that attended private or government clinics, attended to by skilled ANC provider, from wealthiest quintile, had higher education lives in urban area and mostly from South West zone (Table 3). All the factors considered except birth orders, marital status and participation in decision making were statistically associated with having desirable quality of ANC. Similarly, all the factors but marital status affected having minimum acceptable quality of ANC significantly.

**Table 3:** Distribution of respondents receiving good quality ANC and factors associated with quality of ANC services in Nigeria

Characteristics of the Respondents	All women using ANC *n* = 13410	Had Desirable (Good) Quality of ANC	Had minimum acceptable quality of ANC	Determinants of Desirable (Good) Quality of ANC			
				OR(95 % CI)	Sig	aOR(95 % CI)	Sig
Residence							
Urban	6462	*422(6.5)	*1053(16.3)	Reference			
Rural	6948	197(2.8)	458(6.6)	0.44(0.39–0.49)	0.000	0.83(0.74–0.94)	0.002
Educational							
No formal	4080	*139(3.4)	*225(5.5)	Reference			
Primary	3083	127(4.1)	238(7.7)	1.43(1.20–1.70)	0.000	1.57(1.34–1.85)	0.000
Secondary	4991	273(5.5)	704(14.1)	2.56(2.20–2.97)	0.000	2.18(1.86–2.56)	0.000
Higher	1256	80(6.3)	344(27.4)	4.32(3.57–5.22)	0.000	2.69(2.20–3.30)	0.000
Marital status							
Never married	426	14(3.7)	30(7.8)	Reference			
Married or LWSP	12511	576(4.6)	1417(11.3)	1.31(0.99–1.74)	0.062	1.11(0.82–1.52)	0.493
Formerly married	473	29(6.5)	64(14.4)	1.51(1.05–2.17)	0.027	1.36(0.93–2.00)	0.116
Age (29.7,7.1 yrs)							
<20	705	*13(1.8)	*26(3.7)	Reference			
20–29	6131	289(4.7)	660(11.7)	2.12(1.58–2.85)	0.000	1.44(1.09–1.91)	0.011
30–39	5119	241(4.7)	661(12.9)	2.55(1.90–3.43)	0.000	1.63(1.20–2.21)	0.002
40+	1455	77(5.3)	164(11.3)	1.98(1.43–2.76)	0.000	1.53(1.09–2.14)	0.014
Wealth quintile							
Poorest	1406	*26(1.8)	*33(2.3)	Reference			
Poorer	2365	73(3.1)	112(4.7)	1.82(1.34–2.46)	0.000	1.49(1.13–1.95)	0.004
Average	2895	117(4.0)	241(8.3)	2.86(2.15–3.81)	0.000	2.35(1.80–3.06)	0.000
Wealthier	3277	183(5.6)	445(13.6)	4.28(3.25–5.65)	0.000	2.91(2.22–3.82)	0.000
Wealthiest	3467	222(6.4)	680(19.6)	6.47(4.92–8.52)	0.000	3.54(2.65–4.72)	0.000
Geographical zone							
North Central	2125	*51(2.4)	*175(8.2)	Reference			
North East	2018	45(2.2)	139(6.9)	0.88(0.74–1.06)	0.171	1.38(1.16–1.64)	0.000
North West	3275	313(9.5)	400(12.2)	1.40(1.18–1.65)	0.000	1.68(1.42–1.98)	0.000
South East	1630	72(4.4)	195(12.0)	1.11(0.92–1.35)	0.287	0.84(0.70–1.00)	0.052
South South	1566	38(2.4)	167(10.6)	1.17(0.96–1.42)	0.127	0.70(0.59–0.84)	0.000
South West	2796	101(3.6)	435(15.6)	1.47(1.24–1.74)	0.000	0.83(0.71–0.97)	0.023
Birth order							
1	2614	98(3.8)	*309(11.8)	Reference			
2	2349	119(5.1)	302(12.9)	1.24(1.04–1.47)	0.016	1.03(0.88–1.20)	0.710
3	2077	107(5.1)	274(13.2)	1.09(0.91–1.30)	0.354	1.01(0.85–1.19)	0.932
4	6370	296(4.6)	626(9.8)	0.95(0.83–1.10)	0.517	0.98(0.83–1.15)	0.773
Timing of ANC							
1^st^ Trimester	3598	*137(3.8)	*443(12.3)	Reference			
2^nd^ Trimester	8310	433(5.2)	965(11.6)	0.97(0.86–1.09)	0.624	0.92(0.83–1.03)	0.137
3^rd^ Trimester	1453	49(3.4)	104(7.1)	0.67(0.54–0.83)	0.000	0.82(0.67–1.00)	0.053
Number of ANC							
Less than 4 times	2485	*65(2.6)	*137(5.5)	Reference			
4 times or more	10925	555(5.1)	1374(12.6)	2.05(1.73–2.42)	0.000	1.52(1.30–1.79)	0.000
Participates in decision							
Yes	3803	195(5.1)	*379(10.0)	1.12(0.99–1.26)	0.073	-	-
No	9607	424(4.4)	1132(11.8)	Reference			
Skill of ANC							
Skilled	12165	*602(5.0)	*1452(11.9)	Reference			
Unskilled	1245	17(1.4)	59(4.7)	0.33(0.26–0.43)	0.000	0.71(0.57–0.89)	0.003
Place of receiving							
Homes	197	*4(1.1)	*9(2.3)	Reference			
Govt hospital/clinics	377	495(5.3)	1095(11.7)	7.44(3.4–16.28)	0.000	7.04(3.45–14.4)	0.000
Govt others	9339	4(0.9)	13(2.8)	1.63(0.67–3.98)	0.280	3.45(1.53–7.75)	0.003
Private Hospital/Clinic	465	109(3.4)	383(12.6)	6.93(3.15–15.3)	0.000	5.46(2.66–11.2)	0.000
Others	3032	7(3.4)	12(6.0)	4.04(1.6–10.21)	0.003	5.90(2.52–13.8)	0.000
Total	13410	567(4.6)	1487(11.3)				

*significant at *p* = 0.05 chi-square test on independence

The bivariate logistic regression models to determine factors determining receipt of desirable quality of ANC services showed that ANC users’ age at birth of the last child and their place of residence were statistically significant to receiving good quality ANC services. Other significant factors include education attainment, marital status, wealth quintiles to which their households belonged, the geographical zone they lived, the parity, timing of ANC initiation, adequacy of numbers of ANC visits, skill of the ANC provider and the type of ANC facilities visited. The odds of a woman residing in rural area to receive good quality ANC services was 56 % lower than those in urban area (OR = 0.44; 95 % CI: 0.39–0.49). Attendees higher education had four times higher odds of receiving good quality ANC compared to women with no formal education (OR = 4.32; 95 % CI: 3.57–5.22) (Table 3).

The multivariate logistic regression showed that women with higher education had three times higher odds of receiving good quality ANC compared to women with no formal education (OR = 2.69; 95 % CI: 2.20–3.30). In the same pattern, women belonging to households in wealthiest quintile had three and half times highe odds of receiving good quality ANC compared to women in the poorest quintile (OR = 3.54; 95 % CI: 2.65–4.72). The attendees who had 4 or more ANC visits were 50 % times more likely to receive good quality ANC services than those who reported three visits or less (OR = 1.52; 95 % CI: 1.30–1.79). Attendees seen by unskilled health worker were about 30 % less likely to receive good quality ANC services (OR = 0.71; 95 % CI: 0.57–0.89). ANC attendees with higher education were about three times more likely to receive good quality ANC (aOR = 2.69; 95 % CI: 2.20–3.30) as shown in Table 3.

## Discussion

We found that less than 5 % of ANC users in Nigeria received desirable quality of ANC services with about one tenth receiving minimum acceptable quality. Although most attendees made four visits, it was very striking that about one percent of ANC users did not receive any of the ten components considered in this study. This study revealed that the commonest component of ANC offered in Nigeria are measurement of blood pressure and distribution of Iron supplement as they were offered to nearly all the attendees. We identified educational attainment, wealth status, type of health facilities where ANC services was sought and skill acquired by ANC providers as the strongest determinant of receipt of desirable quality of ANC in Nigeria. In addition, place of residence and zones significantly predicted the receipt of good quality ANC in Nigeria.

The use of a nationally representative and population-based data with a large sample size strengthened our findings and made the results very reliable compared to previous studies on quality of ANC services in Nigeria [21, 26, 27, 29]. Considering the vital roles PMTCT plays in reducing incidence and prevalence of HIV/AIDS, the inclusion of health education on PMTCT and HIV counselling and testing and result collection has added value to our study. However, the data used in this study could have suffered recall bias because there was no means of verifying the information provided by the ANC attendees. The data was secondary and collected in a cross sectional setting. Also, we could not ascertain the number of iron and malaria doses received by the ANC attendees. Setting a cut off for acceptable doses could have improved the quality of our findings.

Despite the multi-sectoral efforts to upturn the accessibility and quality of ANC services in Nigeria, the chances of achieving the MDGs [30, 31] is slim. The proportion (<5 %) receiving desirable quality of ANC in Nigeria receiving is much lower than about 23 % obtained in a Nepal study [20]. The Nigeria proportion translates to only one of every 23 ANC attendees receiving desirable quality of ANC. Intuitively, only one of every 40 pregnant women Nigeria receives desirable quality of ANC considering the fact that 33.9 % of pregnant women did not access ANC [11]. It was however strange that one percent of ANC users did not receive any of the 10 components. With the high population in Nigeria, 1 % of over five million annual births [32] translates to approximately 50,000 pregnancies not receiving any care despite visiting ANC providers. However, we found that most of the women that received none of the components attended “homes” for ANC services. Our finding on late ANC commencement agreed with previous studies in Nigeria which opined that the practice could result in missed opportunities of early detection of dangerous fetal and maternal conditions [21, 28].

High proportion of ANC attendees who reportedly attended ANC four or more times in this study was at per with some previous findings in Nigeria and Africa [4, 21, 28]. Our finding on proportions receiving the different components of ANC offered in Nigeria were similar to proportions reported in an earlier studies in Nepal and Nigeria [20, 21].

Unlike a previous study in Nepal [20] that reported about half of the women who had their urine tested for protein and infection and about 45 % which had their blood tested for anaemia, we found about four of every five mothers receiving each of the services. Our findings were also at variance with a previous study in Nigeria that found a low blood and urine test. The study had linked such low practices to lack of capacity of the health centres to perform such tests [26]. Urine and blood analysis are important components of ANC and should be made available in Nigeria considering her high level of health related problems.

The proportion of women who received tetanus toxoid injections and health talks on pregnancy complications were low but similar to report of a Ugandian study but were lower than findings in a Nepal study [20]. Proportion receiving IPD in the current study is much lower than the proportion in a Nepal study [20] and while about two thirds were told about PMTCT of HIV/AIDS only two fifths obtained their HIV status in our study. Knowledge of PMTCT and mothers’ HIV status can help reduce the spread of the epidemic [1, 19, 24]. Malaria remains both a clinical and public health challenge in Nigeria especially among expecting mothers and young children. Despite WHO recommendation and Nigeria government policy that pregnant woman must receive at least two doses of sulfadoxine-pyrimethamine during second and third trimester, we found less than 40 % of ANC attendees receiving the required doses [28].

The strongest determinant of receipt of desirable quality of ANC in Nigeria was type of health facilities the women visited. Government hospitals and health centres as well as private hospitals and clinics have higher odds of rendering desirable quality of ANC than homes or any other ANC provider. We found that skills acquired by ANC providers influenced the quality of ANC offered. This finding was in agreement with outcome of a Nepal study [20]. The WHO recommended minimum of four ANC visits in the course of a pregnancy [1, 9] to obtain optimal care. The adequacy of number of visits paid to ANC providers impacted on receiving desirable quality of ANC. Women with at least four visits had higher likelihood of being given better quality than those with less than four visits. This finding collaborated reports of an earlier Nigeria study [21].

Our finding that pregnant women with higher educational attainment were more likely to be given good quality care was at consonance with outcomes of a recent Nigeria, Uganda and Nepal studies [20, 21]. Women from higher income households were more likely receive good quality ANC in Nigeria. This finding was consistent with previous finding [20]. This outcome is very surprising since ANC care services were free in Nigeria especially in public health facilities. Although it has been established that women of higher socioeconomic status compared to their counterparts in the lower socioeconomic status may have stronger economic power to afford and access better health care and information [4, 14].

Rural–urban differences and geographical location of residence influenced receiving good quality ANC in Nigeria. Mothers in rural areas had lower odds of receiving desirable quality of ANC compared to those in the urban areas. This finding was similar to report of a Nepal study which identified difficulty in getting transported to health care providers as a major problem especially in rural areas [20]. Besides the transport problems, scarcity of health facilities and skilled health providers in rural areas [11] might have worsened the situation in rural Nigeria. We found the factors determining desirable quality of ANC and minimum acceptable quality of ANC to be similar in this study.

## Conclusion

The quality of ANC services is poor and is mostly influenced by women’s residence, age at birth of the last child, education attainment and socio-economic status. Others are the geographical zone they lived, adequacy of numbers of ANC visits, skill of the ANC provider and the type of health facilities where ANC was received. Poor ANC services might negatively impact on the efforts aimed at reducing morbidity and mortality among pregnant women and children. This could make the achievement of three critical MDGs (Goals 5, 6, and 7) a tall dream. It is worrisome that some women attended ANC without receiving any component of ANC. Compliance with WHO recommendations on use of IPD and IPTp for malaria control has been unacceptably poor. Proportion of woman receiving health education on PMTCT and proportion who know their HIV status are abysmally low considering 4.1 % [33] HIV prevalence among pregnant women in Nigeria. The outcomes of this study is a pointer to clinicians, public health practitioners and policy makers not to evaluate success of ANC on the basis of its coverage alone but also on what was offered in the ANC. All stakeholders involved in maternal and child care should do more to ensure that all ANC attendees irrespective of their residence, social or economic status receive all the components of ANC. Pregnant women must be encouraged to commence ANC early, have adequate number of visits and obtain care from skilled. With higher likelihood of quality ANC services been received by the economically better off attendees, are ANC services totally free in Nigeria? Do ANC attendees pay for the components? Could payments be specific to health facilities? It may be necessary to explore the timing and quantity of the components offered the ANC attendees in future studies.

### Strengths and limitations of the study

A nationally representative and population-based data with a large sample size was used for the analysis. Inclusion of health education on PMTCT and women knowing their HIV status have strengthened the study. The data might be affected by recall bias since data was self-reported.
